# NGAL is Downregulated in Oral Squamous Cell Carcinoma and Leads to Increased Survival, Proliferation, Migration and Chemoresistance

**DOI:** 10.3390/cancers10070228

**Published:** 2018-07-10

**Authors:** Javadi Monisha, Nand Kishor Roy, Ganesan Padmavathi, Kishore Banik, Devivasha Bordoloi, Amrita Devi Khwairakpam, Frank Arfuso, Arunachalam Chinnathambi, Tahani Awad Alahmadi, Sulaiman Ali Alharbi, Gautam Sethi, Alan Prem Kumar, Ajaikumar B. Kunnumakkara

**Affiliations:** 1 Cancer Biology Laboratory & DBT-AIST International Laboratory for Advanced Biomedicine (DAILAB), Department of Biosciences & Bioengineering, Indian Institute of Technology Guwahati, Assam 781039, India; j.monisha@iitg.ernet.in (J.M.); r.nand@iitg.ernet.in (N.K.R.); padmavathi@iitg.ernet.in (G.P.); kishore.banik@iitg.ernet.in (K.B.); devivasha@iitg.ernet.in (D.B.); d.amrita@iitg.ernet.in (A.D.K.); 2 Stem Cell and Cancer Biology Laboratory, School of Biomedical Sciences, Curtin Health Innovation Research Institute, Curtin University, Perth, WA 6009, Australia; frank.arfuso@curtin.edu.au; 3 Department of Botany and Microbiology, College of Science, King Saud University, Riyadh 11451, Saudi Arabia; carunachalam@ksu.edu.sa (A.C.); sharbi@ksu.edu.sa (S.A.A.); 4 Department of Pediatrics, College of Medicine, and King Khalid University Hospital, King Saud University, Riyadh 11451, Saudi Arabia; talahmadi@ksu.edu.sa; 5 Department of Pharmacology, Yong Loo Lin School of Medicine, National University of Singapore, Singapore 117600, Singapore; 6 Cancer Program, Medical Science Cluster, Yong Loo Lin School of Medicine, National University of Singapore, Singapore 119228, Singapore; 7 Cancer Science Institute of Singapore, National University of Singapore, Singapore 117599, Singapore; 8 Curtin Medical School, Faculty of Health Sciences, Curtin University, Perth, WA 6009, Australia

**Keywords:** oral cancer, mTOR pathway, secreted glycoprotein, drug resistance

## Abstract

Oral cancer is a major public health burden worldwide. The lack of biomarkers for early diagnosis has increased the difficulty in managing this disease. Recent studies have reported that neutrophil gelatinase-associated lipocalin (NGAL), a secreted glycoprotein, is upregulated in various tumors. In our study, we found that NGAL was significantly downregulated in primary malignant and metastatic tissues of oral cancer in comparison to normal tissues. The downregulation of NGAL was strongly correlated with both degree of differentiation and stage (I–IV); it can also serve as a prognostic biomarker for oral cancer. Additionally, tobacco carcinogens were found to be involved in the downregulation of NGAL. Mechanistic studies revealed that knockdown of NGAL increased oral cancer cell proliferation, survival, and migration; it also induced resistance against cisplatin. Silencing of NGAL activated mammalian target of rapamycin (mTOR)signaling and reduced autophagy by the liver kinase B1 (LKB1)-activated protein kinase (AMPK)-p53-Redd1 signaling axis. Moreover, cyclin-D1, Bcl-2, and matrix metalloproteinase-9 (MMP-9) were upregulated, and caspase-9 was downregulated, suggesting that silencing of NGAL increases oral cancer cell proliferation, survival, and migration. Thus, from our study, it is evident that downregulation of NGAL activates the mTOR pathway and helps in the progression of oral cancer.

## 1. Introduction

Despite significant advancements in the management of oral cancer, it is one of the prime concern worldwide, accounting for approximately 128,000 deaths annually [1,2]. The five-year survival rate of oral cancer is 62.1% (2003–2009); nevertheless, survival rates worsen with advancement in clinical stages (SEER 2003–2009) [3]. Regardless of the unquestionable benefits from the available therapeutic modalities, chemoresistance and recurrence are major complications that reduce the quality of life in patients. This demands the development of novel biomarkers for its early diagnosis and novel targets for the discovery of more potent chemotherapeutic agents for this disease.

Over the past two decades, neutrophil gelatinase-associated lipocalin (NGAL) has received enormous attention in the clinic as a biomarker of kidney injury, cardiovascular injuries, and cancer [4,5,6,7,8]. NGAL, also known as Lipocalin-2 (LCN2), is a 24 kDa glycoprotein in humans encoded by the LCN2 gene located on chromosome 9 at the locus 3p11. Recently, it has emerged as a biomarker for several benign and malignant diseases. Upregulation of NGAL increases the invasiveness of breast, bladder, gastric, gynecological, thyroid, lung, esophageal, colon cancer, and chronic myelogenous leukaemia; however, in pancreatic and oral cancer, it decreases the invasiveness [9,10,11]. Upregulation of NGAL increases cell proliferation of cervical and lung cancer cells, while downregulation reduces cell proliferation [12,13]. NGAL is a well-known modulator of epithelial–mesenchymal transition (EMT), invasion, and migration. Overexpression of NGAL persuades EMT via activation of snail, twist, N-cadherin, fibronectin, MMP-9, and NF-κB; it then upregulates the genes associated with stemness, adhesion, motility, and drug efflux [14,15,16]. Likewise, silencing of NGAL reduces migration and invasion via the downregulation of vimentin, MMP-2, and MMP-9 and increases the expression of E-cadherin [11]. These findings suggest that NGAL plays a key role in the development and progression of cancer. However, the role of NGAL in oral cancer has not been well established thus far. Although several studies have shown that NGAL is downregulated in oral cancer, its expression and role in different types and process of oral cancer development have not been studied thoroughly [17,18]. Therefore, a study on the expression of NGAL in different processes of development of oral cancer can help us to comprehend whether NGAL can serve as a diagnostic and prognostic biomarker for oral cancer.

In the present study, we have examined the expression of NGAL in different stages, grades, tumours from different tissues, degree of differentiation, and different processes of development of oral cancer. We found that NGAL plays a pivotal role in different processes of oral cancer development such as survival, proliferation, invasion, migration, and resistance to chemotherapeutic agents.

## 2. Materials ad Methods

### 2.1. Tissue Microarray

Tissue microarray (TMA) slides for head and neck squamous cell carcinoma (Cat no: HN803b) and oral squamous cell carcinoma (Cat no: OR802) were purchased from US Biomax, Derwood, MA USA.

### 2.2. Immunohistochemistry (IHC)

Expression of NGAL was determined by immunohistochemical analysis. Histostain plus kit (Cat no: 859043, Life Technologies, Carlsbad, CA, USA) was used according to the manufacturer’s protocol. Anti-hNGAL monoclonal antibody was purchased from (Cat no: ab23477, Abcam, Cambridge, MA, USA). The TMAs were deparaffinised and rehydrated using xylene and ethanol and were blocked with 3% hydrogen peroxide in methanol for 30 min. After the antigen retrieval, the sections were incubated in blocking solution for 30 min and then were incubated with primary antibody (1:10 dilution) at 4 °C overnight. The following day, the sections were incubated with secondary antibody for 1 h at room temperature, stained with DAB (3,3′-Diaminobenzidine)counter stained with haematoxylin, and were mounted using DPX.

### 2.3. Scoring

All slides were observed under a Nikon Eclipse Ti-E microscope, and the intensity of immunoreactivity for NGAL was examined. The staining intensity was graded on a scale of 0 to 3+ (0 for no staining; 1+ for weak immunoreactivity; 2+ for moderate immunoreactivity; and 3+ for strong immunoreactivity). The percentage of cells positive for NGAL were graded by the following protocol: grade 0 intensity (<10% positive cells); grade 1+ intensity (10–25% positive cells), grade 2+ intensity (25–50%), grade 3 intensity (50–75% positive cells), and grade 4 intensity (75–100% positive cells). The staining intensity score and the percent immunoreactivity score were then multiplied to obtain a composite score.

### 2.4. Materials

4-(Methylnitrosoamino)-1-(3-pyridinyl)-1-butanone (NNK, Cat No. 78013), *N*′-Nitrosonornicotine (NNN, Cat No. 75285), 4-Nitroquinoline *N*-oxide (4-NQO, Cat No. N8141), Cisplatin (PHR1624), and 5-Flurouracil (5-FU) (F6627) were purchased from Sigma-Aldrich, Saint Louis, MO, USA.

### 2.5. Cell Culture

Human squamous cell carcinoma of the tongue, SAS cells were procured from Rajiv Gandhi Centre for Biotechnology (RGCB), Trivandrum, India. These cells were maintained in Dulbecco’s Modified Eagle Medium (DMEM; Gibco™; Life Technologies) supplemented with 10% fetal bovine serum (FBS; Gibco^®^, Grand Island, NY, USA) and 1× Penstrep (Invitrogen, Carlsbad, CA, USA). The cells were cultured and maintained at 37 °C in 5% CO_2_ and 95% humidity.

### 2.6. Antibodies

S6 Ribosomal protein (dilution 1:2000; Cat No. 2317S), Phospho-S6 Ribosomal protein (Ser235/236) (dilution 1:2000; Cat No. 4858T), LC3B (dilution 1:1000; Cat No. 2775S), Caspase-9 (dilution 1:1000; Cat No. 9508T), Bcl-2 (dilution 1:1000; Cat No. 15071), MMP-9 (dilution 1:1000; Cat No. 13667P), and cyclin D1 (dilution 1:1000; Cat No. 2978BC), GAPDH (dilution 1:2000; Cat No. 2118S) were purchased from Cell Signaling Technology, Danvers, MA, USA. Antibodies against NGAL (dilution 1:3000; Cat No. ab23477), anti-mouse secondary antibody (dilution 1:6000; Cat No. ab97040), and anti-rabbit secondary antibody (dilution 1:6000; Cat No. ab97080) were purchased from Abcam.

### 2.7. shNGAL Knockdown

shRNA-mediated knockdown of NGAL was carried out in the SAS cell line. Human shNGAL plasmids (Table 1) and puromycin (Cat No. P8833, Sigma-Aldrich) were purchased from Sigma. SAS cells were seeded at a concentration of 25 × 10^4^ cells/well in 1 mL of medium in a 24-well plate. The next day, cells were transfected with shRNA control and shNGAL plasmids (2 µg of DNA) using X-treme gene 9 DNA transfection reagent (Cat No. 06365787001, Sigma-Aldrich) for 48 h. The medium containing transfection reagent was replaced with fresh DMEM medium and the cells were allowed to recover for 24 h. Then SAS cells were selected with 1μg/mL puromycin and knockdown clones were established.

### 2.8. Cell Viability

Briefly, 2 × 10^3^ cells/well were seeded in 96-well plates in sextuplicate and incubated for 24 h and 48 h time points. After each time point, 10 µL of 3-(4,5-dimethylthiazol-) 2,5-diphenyl tetrazolium bromide (MTT) (5 mg/mL; Cat No. M2128, Sigma-Aldrich) was added to the cells and was further incubated for 2 h at 37 °C. The MTT solution was removed and 100 μL of DMSO (Cat No. 1.16743.0521, Merck, Darmstadt, Germany) was added to each well and the absorbance was then measured at 570 nm using an Infinite M200 Pro (Tecan Group Ltd., Männedorf, Switzerland) after 1 h.

### 2.9. Cell Cycle Analysis

Control shRNA and shNGAL cells were plated at a density of 1 × 10^5^ cells/well, and, after 24 h, cells were trypsinized, washed with phosphate-buffered saline (PBS), and fixed with 75% ethanol at −20 °C overnight. The following day, cells were washed with PBS, treated with PI/RNase solution (Cat No. A35126, Invitrogen) for 20 min in the dark and analyzed using a flow cytometer (FACS Calibur, Becton-Dickinson, Franklin Lakes, NJ, USA). 25,000 cells in each sample were analyzed to obtain a measurable signal. The data were analyzed on FCS express6 (De Novo Software, Glendale, CA, USA).

### 2.10. Cell Survival Assay

Control shRNA and shNGAL cells were seeded in a 6-well plate at a density of 1 × 10^3^ cells/well. The cells were grown for fifteen days, then colonies were fixed with 70% ethanol and were stained with crystal violet. Pictures of individual wells were taken and were analyzed using imageJ 1.x software [19], and the surviving fraction was calculated.

### 2.11. In Vitro Wound Closure Assay

Control shRNA and shNGAL cells were seeded in 6-well plates and were allowed to grow till confluency, and then serum starved for 8 h. Confluent monolayers were scratched with a pipette tip. Plates were washed with PBS to remove non-adherent cells, and the wound was photographed at 0 h and 8 h, respectively. The percentage of wound area was calculated.

### 2.12. Cell Invasion and Migration Assay

Control shRNA and shNGAL cells were serum starved for 18 h before seeding onto transwell inserts (Cat No. 3422, Corning, New York, NY, USA) pre-coated with matrigel. Following serum starvation, the cells were trypsinized and were seeded at a concentration of 5 × 10^4^ cells in the upper chamber of the transwell insert; in the lower chamber, medium containing 10% FBS was added as a chemo-attractant. Cells were then incubated for another 24 h at 37 °C. The migrated cells at the bottom of the transwell insert were fixed in 70% ethanol and were stained with crystal violet solution. Stained cells were visualized under an inverted microscope and photographs were taken using a Nikon 500 camera. After the photographs were taken, the membrane was dissolved in 1% sodium dodecyl sulphate (SDS) (Cat. No. 436143, Sigma-Aldrich) solution at 37 °C for 1 h and absorbance was read at 595 nm in a Tecan plate reader.

### 2.13. RNA Isolation and Reverse Transcriptase PCR

Total RNA was extracted using TRIzol reagent (Invitrogen), and cDNA synthesis was carried out using High-Capacity cDNA Reverse Transcription Kit (Life Technologies). PCR was then performed with 1 μL of cDNA as a template. Primer sequences and amplicon lengths are listed in Table 2.

### 2.14. Western Blot Analysis

Whole cell lysates were prepared by lysing the cells in whole cell lysis buffer (20 mM HEPES, 2 mM EDTA, 250 mM NaCl, 0.1% NP-40) in the presence of protease inhibitors (2 µg/mL Leupeptin hemisulfate, 2 µg/mL Aprotinin, 1 mM PMSF, 1 mM DTT). The protein concentration of the lysates was measured using the Bradford assay (Cat No. 500-0205; Bio-rad, Hercules, CA, USA) and 50 µg of protein was mixed with 5× Laemmli Buffer (250 mM TrisHCl, 10% SDS, 30% Glycerol, 5% β-mercaptoethanol, 0.02% Bromophenol blue), electrophoresed in a 12% SDS-acrylamide gel, and transferred to nitrocellulose transfer membrane (Bio-rad). The membranes were blocked with 5% non-fat milk in tris-buffered saline (TBS: 0.2 M Tris base, 1.5 M NaCl, H_2_O) containing 1% tween 20 (TBST). The blots were probed with appropriate primary antibodies overnight. The following day, the blots were washed with TBST, were incubated in appropriate horseradish peroxidase-conjugated secondary antibody, and were visualized using an Optiblot ECL Detection Kit (Cat No. ab133406, Abcam). β-actin/GAPDH was used as the loading control.

### 2.15. Propidium Iodide Flow Cytometry (PI/FACS)

The cell death induced by chemotherapeutic agents was determined by staining with propidium iodide (PI) (conct. 1 mg/mL; Cat No. P4170, Sigma-Aldrich). Control shRNA and shNGAL cells were seeded in a 6-well plate at a density of 5 × 10^4^ cells/well. After 24 h, the cells were treated with different concentrations of cisplatin and 5-Flurouracil for 48 h. After 48 h, the cells were harvested and were washed with PBS twice. 10 μL of PI was added and was analyzed by flow cytometry (FACSCalibur, Becton-Dickinson). The data were analyzed using FCS Express 6 software.

### 2.16. Statistical Analysis

All the statistical analysis was carried out using Student’s *t*-test or one-way ANOVA followed by Tukey test [20]. *p*-value less than 0.05 was considered as statistically significant.

## 3. Results

To understand the role of NGAL in oral cancer, we carried out immunohistochemical analysis in oral cancer patient tissues. The tissue microarray contained tissues of different premalignant lesions, stages, grades, tissues, and degree of differentiation of oral cancer. Next, we examined the effect of potent tobacco carcinogens such as NNK, NNN, and the synthetic oral carcinogen 4-NQO on the expression of NGAL in oral cancer cells. Then, we established the role of NGAL on different hallmarks of cancer and elucidated the mechanisms involved.

### 3.1. NGAL Expression Was Found to Be Downregulated in Oral Cancer

**Figure 1:**
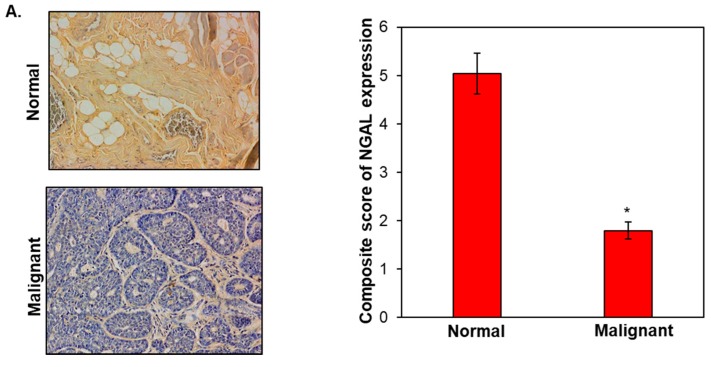

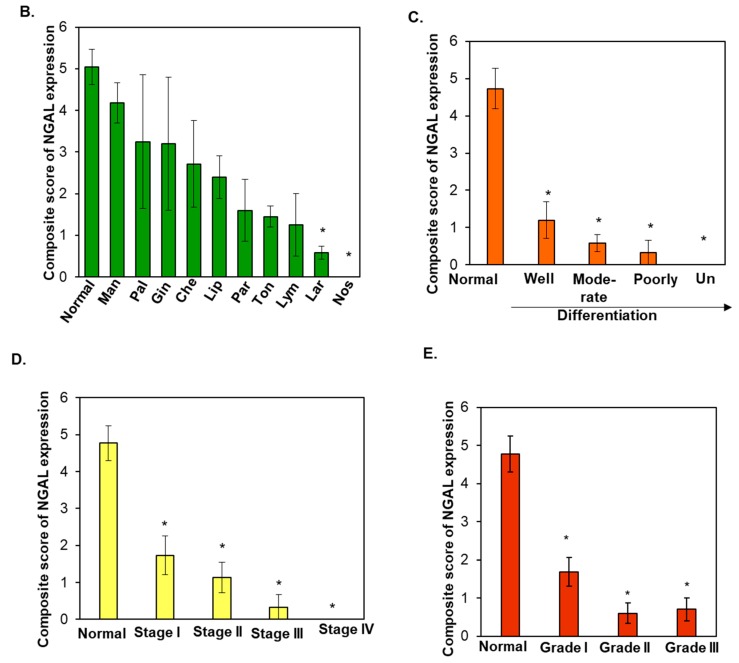
Expression of NGAL (neutrophil gelatinase-associated lipocalin) in oral cancer. (**A**) Representative images of expression of NGAL in oral cancer (left panel). Expression of NGAL in normal (no. of samples (*n*) = 21) vs. malignant (*n* = 139) oral cancer tissues (right panel). (**B**) Expression of NGAL in different tissues of oral cancer. Lar: Larynx, Nos: Nose, Ton: Tongue, Che: Cheek, Gin: Gingiva, Lym: Lymph node, Man: Mandible, Par: Parotid gland, Pal: Palate. (**C**) Expression of NGAL with degree of differentiation of oral cancer. (**D**) Expression of NGAL in different stages of oral tongue cancer tissues. (**E**) Expression of NGAL in different grades of oral tongue cancer tissues. Data are mean ± SE. * = *p* < 0.05.

To understand the role of NGAL in oral cancer, we first determined the expression of NGAL in oral cancer tissues. Our results showed moderate expression of NGAL in normal tissues compared to weak to moderate expression in malignant tissues (Figure 1A). Intriguingly, the majority of the well-differentiated epithelial cells of both malignant and normal tissues showed moderate expression of NGAL. Expression of NGAL was observed in all the tumours arising from the oral cavity, including mandible, cheek, gingiva, lip, palate, parotid gland, tongue, lymph node, and larynx, and was found to be downregulated (Figure 1B). Weak to moderate staining of NGAL was observed in the above-mentioned tissues, except the nose, where it was negative. Moreover, the expression of NGAL was inversely associated with the degree of differentiation of tumours. Normal- and well-differentiated tongue tissues showed positive staining of NGAL in comparison to very weak positive staining observed in moderately differentiated and poorly differentiated tongue tissues (Figure 1C). However, no positive expression was observed in the undifferentiated tongue cancer tissues. This suggests that NGAL can serve as a prognostic biomarker for oral cancer. The expression of NGAL also correlated with different stages of tongue cancer tissues where stage I showed high expression and stage IV negative expression in comparison to normal tissues. Similarly, the expression of NGAL was downregulated significantly with an increase in grade of oral cancer in comparison to normal tissues (Figure 1D,E). Furthermore, NGAL was also downregulated in different processes and pathological types of oral cancer and was strongly associated with lymph node metastases (Supplementary Figures S1–S3).

### 3.2. Tobacco Components Downregulated the Expression of NGAL

**Figure 2:**
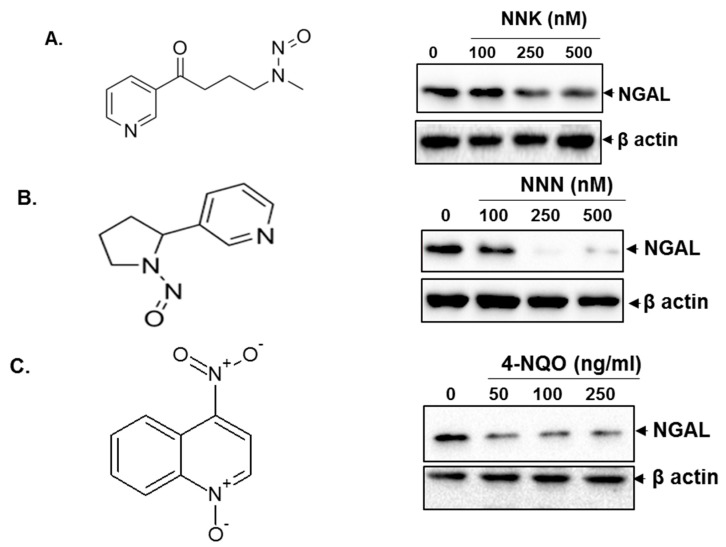
Tobacco components downregulated the expression of NGAL in oral cancer cell line SAS. (**A**) Structure of NNK (left panel). Western blot analysis of expression of NGAL after treatment with NNK for 48 h in SAS cells (*n* = 2) (right panel). (**B**) Structure of NNN (left panel). Western blot analysis of expression of NGAL after treatment with NNN for 48 h in SAS cells (*n* = 2) (right panel). (**C**) Structure of 4-NQO (left panel). Western blot analysis of expression of NGAL after treatment with 4-NQO for 48 h in SAS cells (*n* = 2) (right panel).

NGAL is downregulated in oral cancer tissues and it is well established that tobacco is the prime risk factor for oral cancer [17,21]. Therefore, we determined whether tobacco carcinogens are involved in the downregulation of NGAL. We treated SAS cells with different concentrations of NNK (Figure 2A), NNN (Figure 2B), and the synthetic carcinogen 4-NQO (Figure 2C) and observed that these tobacco components downregulated the expression of NGAL in a dose-dependent manner. This suggests that tobacco carcinogens play a key role in regulating the expression of NGAL.

### 3.3. Silencing of NGAL Increased Proliferation and Survival of Oral Cancer Cells

**Figure 3:**
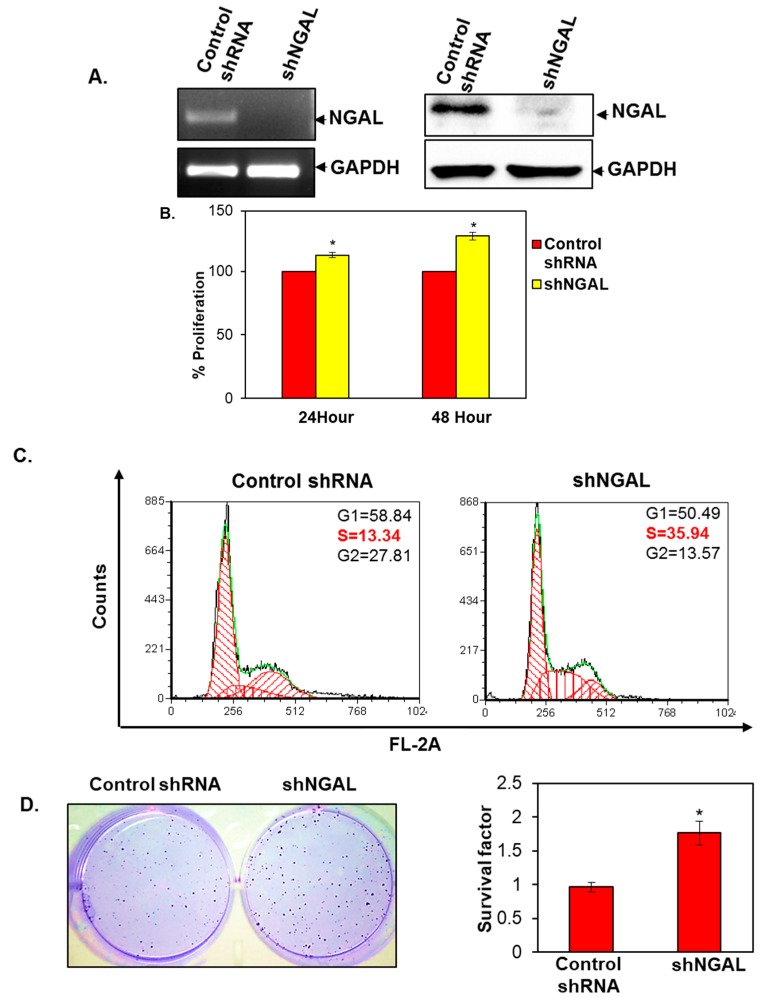
Silencing of NGAL in oral cancer cells. (**A**) qRT–PCR showing the mRNA expression of NGAL in SAS cells post knockdown (left panel). Western blot analysis showing the expression of NGAL in SAS cells post knockdown (right panel). (**B**) Percentage increase in cell viability of control shRNA and shNGAL cells, determined by MTT assay (*n* = 2). (**C**) Cell cycle distribution was determined by flow cytometric analysis in control shRNA and shNGAL cells (*n* = 3). (**D**) Clonogenic assay showing an increase in number of colonies (left panel). Graphical representation of increase in number of colonies in NGAL knockdown cells (*n* = 2) (right panel). Data are means ± SE. * = *p* < 0.05.

The fundamental property of cancer cells is to sustain cell survival and proliferation. Therefore, we sought to study the effect of silencing of NGAL on the proliferation and survival of oral cancer cells. To study the role of NGAL in oral cancer cell proliferation and survival, we silenced the expression of NGAL (Figure 3A). We carried out an MTT assay and observed that knockdown of NGAL increased cell viability in a time-dependent manner (Figure 3B). To confirm that knockdown of NGAL increases cell viability, we studied its effect on different phases of the cell cycle. We found that silencing of NGAL led to an increase in the number of cells in S-phase and reduced the number of cells in G2/M phase in comparison to control shRNA (Figure 3C). The increase in number of cells in S-phase suggests that NGAL knockdown allows cancer cells to proliferate uninterruptedly and pass through the G2/M check point. In addition, in NGAL deficient cells, we observed that the expression of cyclin D1 is upregulated, which is regulated by the NF-κB/PI3K/mTOR pathways [22,23]. We also assessed if knockdown of NGAL increases oral cancer cell survival by using a clonogenicity assay (Figure 3D). We observed a two-fold increase in the number of colonies in the shNGAL group in comparison to control shRNA group.

### 3.4. Silencing of NGAL Increases Invasion and Migration of Oral Cancer Cells

**Figure 4:**
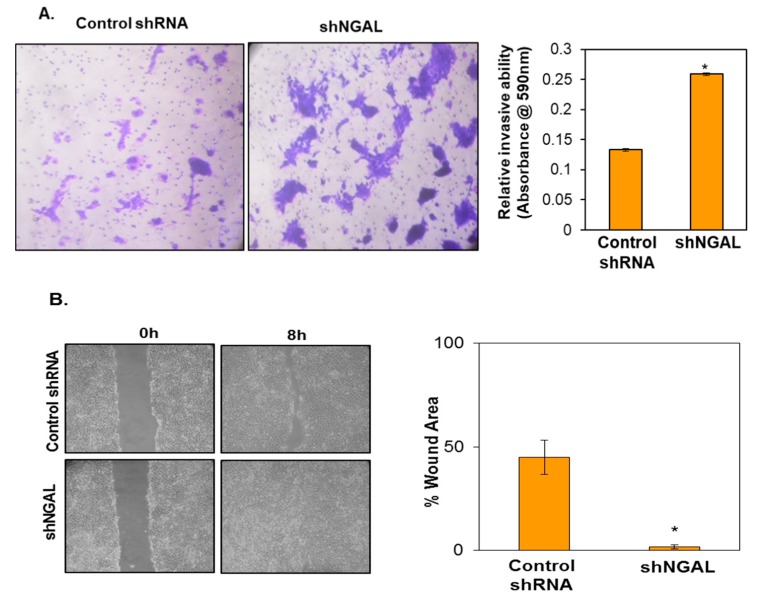
Silencing of NGAL increased the invasion and migration of oral cancer cells. (**A**) Cell invasion was determined by a transwell invasion assay. Cells invading through the matrigel were fixed, stained, and photographed under an inverted microscope at a 20× magnification. Graphical representation of increase in cells invading the lower surface of transwell insert (right panel). (**B**) Cell migration was detected by scratch wound healing assay. Photographs were taken at 10× magnification. Graphical representation of decrease in wound area (right panel). Data are means ± SE. * = *p* < 0.05 (*n* = 4).

Our IHC results advocate that downregulation of NGAL is strongly associated with metastases; accordingly, we hypothesized that knockdown of NGAL may induce invasion and migration of oral cancer cells. To confirm this, we performed in vitro invasion and migration assays using NGAL knockdown cells. Results from the transwell migration assay suggested that the NGAL knockdown cells possessed higher invasive ability than shRNA control cells. The number of cells that invaded the lower part of the transwell insert was higher in the case of shNGAL cells in comparison to control cells (Figure 4A). Similarly, in the in vitro wound healing assay, the wound was healed within 8 h in the case of shNGAL cells in comparison to control cells. This indicates that shNGAL cells have higher migratory potential (Figure 4B). Similarly, in NGAL silenced cells, MMP-9 was found to be upregulated, which might be responsible for the increase in cell motility.

### 3.5. Silencing of NGAL Activates mTOR Signalling and Suppresses Autophagy

**Figure 5:**
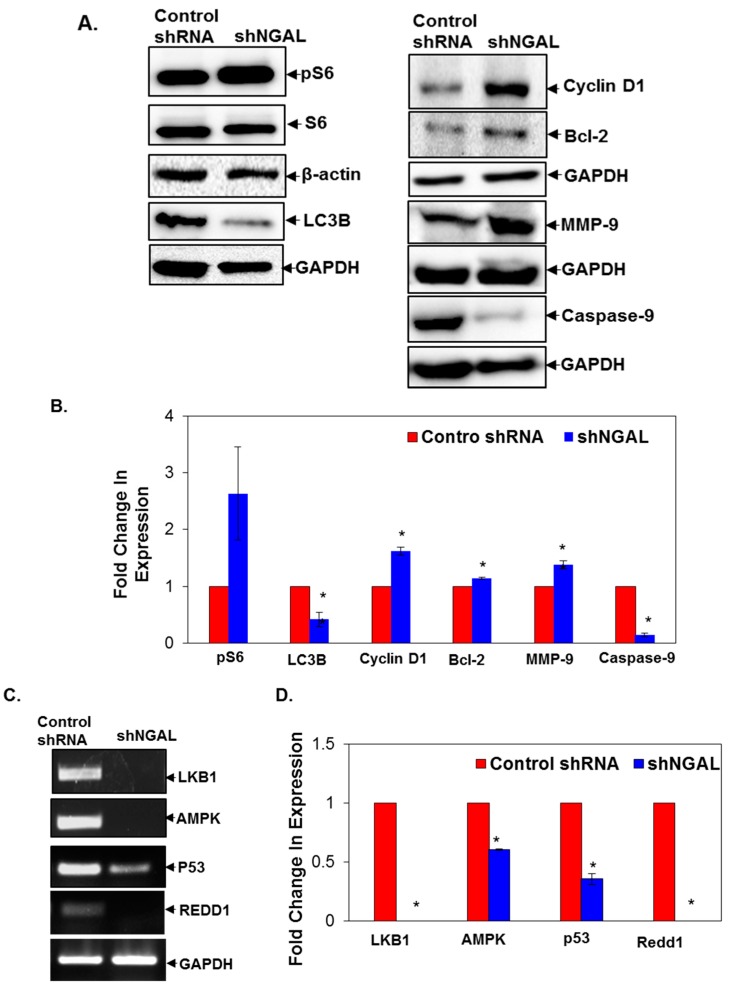
Silencing of NGAL activated mTOR signalling and induced autophagy. (**A**) Expression of proteins involved in mTOR signalling and autophagy. (**B**) Fold change in expression of proteins as analyzed by image lab software (*n* = 2). (**C**) mRNA expression of LKB1-AMPK-P53-Redd1 in NGAL knockdown cells. (**D**) Fold change in mRNA expression as analyzed by image lab software (*n* = 3). Data are means ± SE. * = *p* < 0.05.

Our previous results suggest that tobacco components downregulated the expression of NGAL, and loss of NGAL increased oral cancer cell proliferation, survival, invasion, and migration. However, the underlying mechanism is not clear. Increasing evidences suggest that tobacco components play a key role in the development of oral cancer and are known to regulate the Akt/mTOR pathway. Therefore, we studied the effect of silencing of NGAL on the activation of S6, a well-established marker of the mTOR pathway. We observed that knockdown of NGAL activated S6 (serine 235/236) (Figure 5A,B). Recently, Dowling et al., 2007 reported that metformin inhibited the activation of S6 via the AMP-activated protein kinase (AMPK) pathway [24]. Hence, we studied the expression of AMPK in NGAL-silenced cells and observed that the expression of AMPK was downregulated, indicating that AMPK is the intermediate link between NGAL and S6. LKB1 is upstream of AMPK, and, as AMPK is the only substrate, we analyzed the expression of LKB1 and found that it was downregulated [25]. Thus, knockdown of NGAL activates mTOR signalling via the AMPK-LKB pathway. Reports suggest that, during hypoxia or energy stress in the head and/or neck, squamous cell carcinoma (HNSCC) cells, regulated in developmentand DNA damage responses -1 (Redd1) inhibits mTOR signalling by upregulating AMPK [26,27]. Hence, we studied the expression of Redd1 in NGAL knockdown cells and found that the expression of Redd1 is completely inhibited (Figure 5C,D). Besides Redd1, AMPK is also known to regulate and activate p53 during metabolic stress [28]. Thus, p53 serves as a downstream target of AMPK, and we found that in NGAL knockdown cells the expression of p53 was found to be downregulated (Figure 5C,D).

Moreover, the promoter region of Redd1 is known to possess the consensus p53 family binding element that is required for regulation of Redd1 by p53 [26,29]. This suggests that Redd1 is a direct transcriptional target of p53 and can be a connecting link between AMPK and Redd1. Therefore, silencing of NGAL increases survival, proliferation, invasion, and migration of oral cancer cells via the LKB1-AMPK-p53-Redd1-mTOR axis (Figure 5C,D). We observed that knockdown of NGAL upregulated cyclin D1, Bcl-2, and MMP-9 as well as downregulated caspase-9, confirming the same (Figure 5A,B). In addition to the significant role of mTOR in cancer progression, activation of mTOR downregulates autophagy [30,31,32]. Thus, we studied the expression of LC3B, an autophagy marker, and observed that the expression of LC3B was found to be downregulated. This suggests that NGAL-silenced cells are more resistant to autophagy-induced cell death, and decreased autophagy provides a survival advantage. Overall, our results suggest that NGAL knockdown cells were more resistant to autophagy, which was mediated via the LKB1-AMPK-p53-Redd1 axis and activation of mTOR signalling.

### 3.6. Silencing of NGAL Selectively Induces Resistance Against Cisplatin

**Figure 6:**
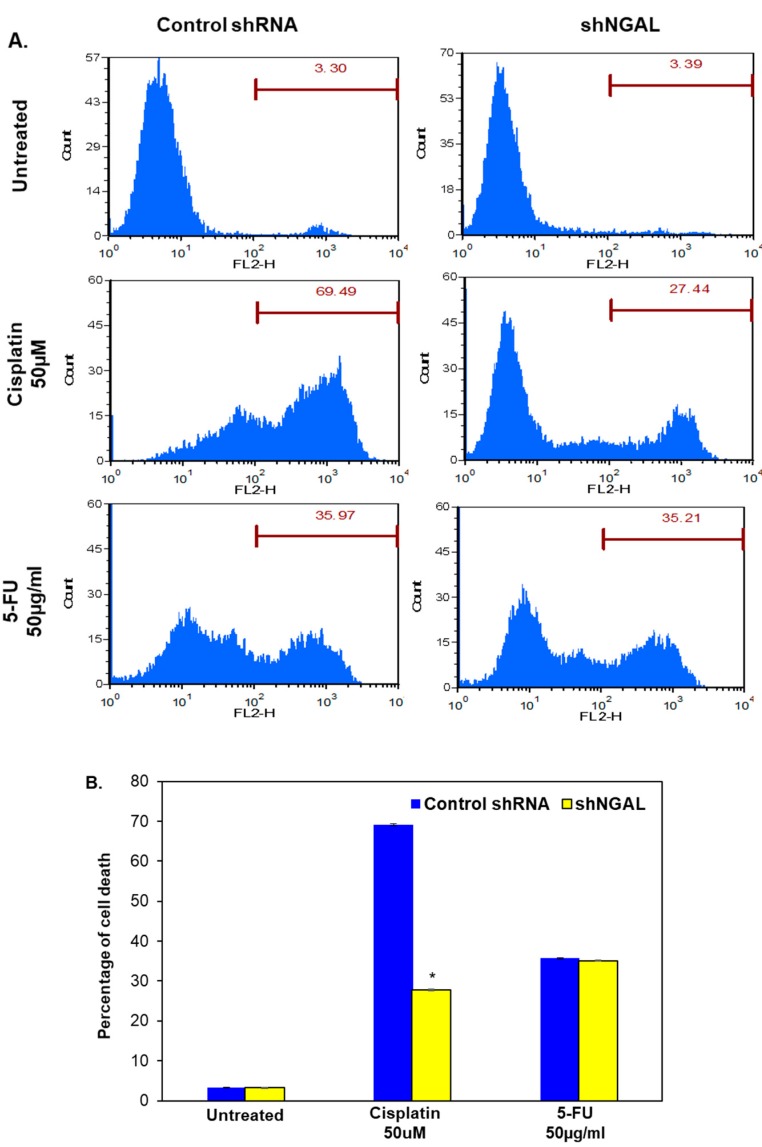
Silencing of NGAL selectively induces resistance against cisplatin. (**A**) Cells were treated with cisplatin and 5-FU, and percentage of cell death was measured by staining with propidium iodide on flowcytometry at 48 h. (**B**) Graphical representation of percentage of cell death (*n* = 3). Data are means ± SE. * = *p* < 0.05.

Development of resistance is the major reason for the failure of chemotherapeutic agents in the clinic. Therefore, we studied the role of NGAL in the development of resistance against the first-line therapeutic agents, cisplatin and 5-FU. We observed that knockdown of NGAL selectively induced resistance against cisplatin, while both control shRNA and shNGAL cells were sensitive to 5-FU (Figure 6A,B). Upregulation of cyclin D1 and Bcl-2 as well as downregulation of caspase-9 might be the reason for the development of chemoresistance. However, the mechanism requires further study.

## 4. Discussion

We studied the expression of NGAL in oral cancer tissues and found that NGAL was downregulated in primary tumour and metastatic tissues. Our results were consistent with previous studies where NGAL was found to be downregulated in oral cancer tissues [17,18]. Downregulation of NGAL was found to be strongly correlated with the degree of differentiation and stage of oral cancer. Similarly, the study carried out by Hiromoto et al., 2011, showed that the downregulation of NGAL was associated with the degree of differentiation of tumours [17]. Thus, NGAL can serve as biomarker for identifying the degree of differentiation, prognosis, and severity of the disease. However, there are no reports about the expression of NGAL with respect to age, tissues, stages, grades, etc. in oral cancer. The expression of NGAL was found to be downregulated in malignant tongue, larynx, lip, cheek, gingiva, and palatal tissues of the oral cavity. Moreover, downregulation of NGAL was evident in all the stages (stage I–IV) and grades (grade I–III) of oral squamous cell carcinoma (OSCC).

Because tobacco is a well-characterized risk factor for oral cancer, we investigated whether NGAL is regulated by tobacco carcinogens. The main carcinogens characterized from tobacco smoke include benzo[a]pyrene, nicotine, NNK, NNN, dibenzo[a]pyrene, benzene, nitrobenzene, 2-toluidine, and 2-6-dimethylaniline. Upon activation, NNK and NNN induce mutations in tumour suppressor genes and oncogenes; they form DNA adducts that result in tumour initiation [33,34,35,36,37]. 4-NQO is a synthetic tobacco carcinogen used to induce oral cancer in mouse that mimics the oral cancer development in humans [38,39]. In our study, it was found that tobacco carcinogens NNK, NNN, and 4-NQO downregulated the expression of NGAL in a dose-dependent manner. This indicates that NGAL plays a key role in tobacco-induced carcinogenesis.

Next, we found that downregulation of NGAL induced oral cancer cell proliferation, survival, invasion, and migration. Many studies report that NGAL plays a key role in the invasion and migration of oral cancer and other cancers. Recently, Lin et al., 2016 reported that knockdown of NGAL increased in vitro cell motility and in vivo metastases [18]. However, this is the first study that has shown that knockdown of NGAL increases in vitro cell viability and survival in oral cancer. Similar to our findings, a recent study in colorectal cancer showed that knockdown of NGAL increased cell proliferation, survival and induced EMT [40]. Presently, the mechanism involved requires further study. Our study shows that knockdown of NGAL activated mTOR signalling and reduced autophagy via the LKB1-AMPK-p53-Redd1 signalling axis. Aberrant activation of mTOR is seen in OSCC and is associated with poor prognosis [41,42,43,44,45]. Phosphorylated S6, the downstream target of mTOR, was found to be upregulated in epithelial dysplasia and OSCC; it also can serve as a potent diagnostic biomarker for oral cancer [46]. mTOR signalling can be activated by various stimuli. During hypoxia or energy starvation, LKB1 is activated, which, in turn, phosphorylates AMPK. Thus, the activated AMPK phosphorylates TSC2, which results in switching off mTOR signalling [47,48]. It is well established that activation of mTOR signalling inhibits autophagy, and studies also suggest that Redd1 regulates autophagy [49]. A similar mechanism was observed in our study, indicating that silencing of NGAL mediates autophagy via Redd1. Moreover, as mentioned earlier, p53 was found to be downregulated in NGAL knockdown cells. Our results were similar to previous studies in which NGAL was shown to regulate the expression of p53 [50,51].

Furthermore, AMPK activates p53 during metabolic stress by phosphorylating MDMX on serine 34, resulting in inhibition of p53 ubiquitylation [28]. Moreover, in cells lacking p53, ectopic expression of p53 induced the endogenous activity of Redd1; additionally, the promoter region of Redd1 comprises of p53 binding sites, indicating that Redd1 is a direct transcriptional target of p53 [26]. Thus, our study demonstrates that knockdown of NGAL activates the mTOR pathway via the LKB1-AMPK-p53-Redd1 signalling axis. Moreover, the expression of cyclin-D1, Bcl-2, and MMP-9 were upregulated and caspase-9 was downregulated, which are the key molecules involved in oral cancer cell proliferation, survival, invasion, and migration. In addition to promoting mTOR signalling, knockdown of NGAL decreased autophagy. Activation of autophagy by many chemotherapeutic agents in HNSCC induced apoptosis and downregulated the mTOR pathway. Many small molecule tyrosine kinase inhibitors such as gefitinib, erlotinib, and dasatinib induced autophagy and suppressed mTOR signalling, indicating that an increase in autophagy suppresses tumour growth in vitro and in vivo [52]. These studies indicate that autophagy serves as a tumour suppressor. Thus, our study clearly demonstrates that knockdown of NGAL increases oral cancer cell proliferation, survival, invasion, and migration by upregulating mTOR signalling and suppressing autophagy.

## 5. Conclusions

**Figure 7:**
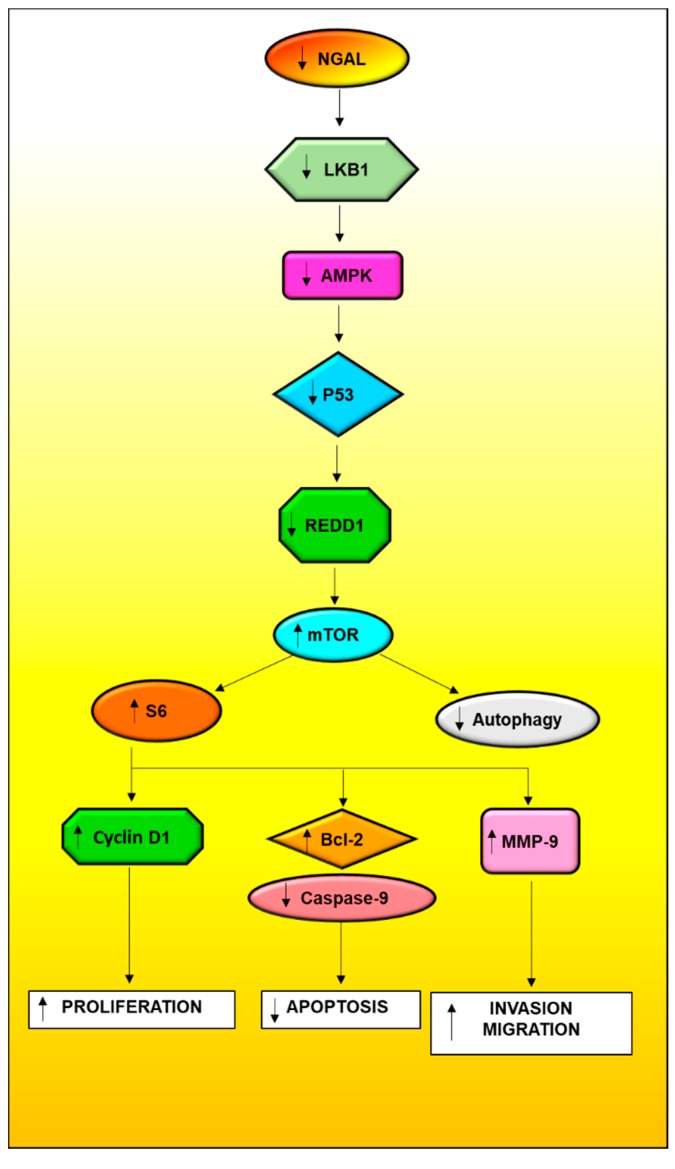
Downregulation of NGAL activates mTOR signaling via LKB1-AMPK-p53-Redd1 and decreases autophagy.

Our results suggest that NGAL is downregulated in oral cancer tissues and is strongly associated with degree of differentiation, stage of the tumour, and lymph node metastases. The tobacco components, primarily NNK, NNN, and the synthetic carcinogen 4-NQO, were implicated in the downregulation of NGAL. Mechanistic studies revealed that knockdown of NGAL augmented cell survival, invasion, and migration by activating the mTOR pathway and also downregulated autophagy via the LKB1-AMPK-p53-Redd1 signalling axis (Figure 7). This suggests the NGAL is one of the key molecule involved in oral cancer tumorigenesis. Therefore, agents that can restore the expression of NGAL would be advantageous in developing effective therapies against this dreadful disease.

## Figures and Tables

**Table 1:** Control shRNA (SHC204) and NGAL shRNA sequences used for transfection.

S. No	Clone	Sequence
1	TRCN0000372769	CCGGCAATTCTCAGAGAAGACAAAGCTCGAGCTTTGTCTTCTCTGAGAATTGTTTTTG
2	TRCN0000378896	CCGGGAGTGGTGAGCACCAACTACACTCGAGTGTAGTTGGTGCTCACCACTCTTTTTG
3	TRCN0000372827	CCGGGGAGCTGACTTCGGAACTAAACTCGAGTTTAGTTCCGAAGTCAGCTCCTTTTTG
4	TRCN0000060288	CCGGGCTGGGCAACATTAAGAGTTACTCGAGTAACTCTTAATGTTGCCCAGCTTTTTG
5	TRCN0000060289	CCGGCCAGCATGCTATGGTGTTCTTCTCGAGAAGAACACCATAGCATGCTGGTTTTTG
6	SHC204	CCGGCGTGATCTTCACCGACAAGATCTCGAGATCTTGTCGGTGAAGATCTTTTT

**Table 2:** List of primers and their sequences used to study mRNA expression.

Gene		Primers	Tm (°C)	Amplicon Size
NGAL	F	5′ATGCCCCTAGGTCTCCTGT3′	55 °C	597 bp
	R	5′TCAGCCGTCGATACACTG3′		
LKB1	F	TCAAAATCTCCGACCTGGGC	55 °C	570 bp
	R	TGTGACTGGCCTCCTCTTCT		
AMPK	F	CGGCAAAGTGAAGGTTGGCAA	59 °C	227 bp
	R	CAAATAGCTCTCCTCCTGAGAC		
P53	F	CTGCCCTCAACAAGATGTTTTG	55 °C	172 bp
	R	CTATCTGAGCAGCGCTCATGG		
Redd1	F	CTGATGCCTAGCCAGTTGGT	55 °C	233 bp
	R	GAGCTAAACAGCCCCTGGAT		
GAPDH	F	AGGTCGGAGTCAACGGATTTG	60 °C	532 bp
	R	GTGATGGCATGGACTGTGGT		

## Supplementary Materials

The following are available online at http://www.mdpi.com/2072-6694/10/7/228/s1, Figure S1: Expression of NGAL in different stages of the development of oral cancer. CAT: Cancer adjacent tissue. Mean composite score of NGAL levels in Normal tissues (*n* = 10), inflammation (*n* = 5), hyperplasia (*n* = 5), cancer adjacent tissues (*n* = 5), malignant tissues (*n* = 42), and metastatic tissues (*n* = 4). Data are mean ± SE. * = *p* < 0.05. Figure S2: Expression of NGAL in different types of oral cancer. Mean composite score of NGAL levels in NT-Normal tissue (*n* = 10), MEC-Mucoepidermoid carcinoma (*n* = 8), ACC-Adenoid cystic carcinoma (*n* = 3), BCC-Basal cell carcinoma (*n* = 2), SCC-Squamous cell carcinoma (*n* = 28), and MSCC-Metastatic squamous cell carcinoma (*n* = 4) of oral cancer. Data are mean ± SE. * = *p* < 0.05. Figure S3: Expression of NGAL with age and gender in oral cancer tissues. (A) Mean composite score of NGAL levels in tissues from normal male (*n* = 11), normal female (*n* = 10), malignant male (*n* = 100), and malignant female (*n* = 39) patients with oral cancer. (B) Mean composite score of NGAL levels in normal (*n* = 17) and malignant oral cancer patient tissues of age groups 0–30 year (*n* = 4), 30–45 year (*n* = 18), 45–60 year (*n* = 63), and 60–100 year (*n* = 52). Data are mean ± SE. * = *p* < 0.05.
