# Supplementary material for: NGAL is Downregulated in Oral Squamous Cell Carcinoma and Leads to Increased Survival, Proliferation, Migration and Chemoresistance
Source: Cancers (Basel). 2018 Jul 10;10(7):228. doi: 10.3390/cancers10070228 (PMC6071146; doi:10.3390/cancers10070228)
Supplement: Supplementary file 1 [file cancers-10-00228-s001.pdf]

Supplementary Materials: NGAL is downregulated in oral squamous cell carcinoma and leads to increased survival, proliferation, migration and chemoresistance

Javadi Monisha, Nand Kishor Roy, Ganesan Padmavathi, Kishore Banik, Devivasha Bordoloi, Amrita Devi Khwairakpam, Frank Arfuso, Arunachalam Chinnathambi, Tahani Awad Alahmadi, Sulaiman Ali Alharbi, Gautam Sethi, Alan Prem Kumar Ajaikumar B. Kunnumakkara

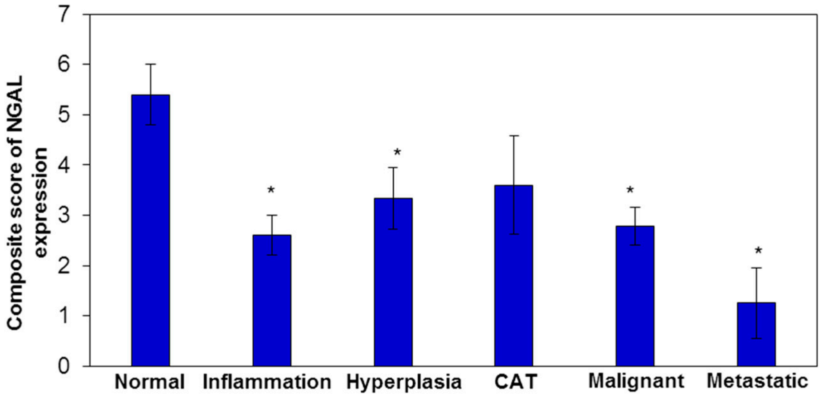

Figure S1. Expression of NGAL in different stages of the development of oral cancer. Mean composite score of NGAL levels in Normal tissues ($n = 10$), inflammation ($n = 5$), hyperplasia ($n = 5$), cancer adjacent tissues CAT ($n = 5$), malignant tissues ($n = 42$), and metastatic tissues ($n = 4$). Data are mean ± SE. * = $p < 0.05$.

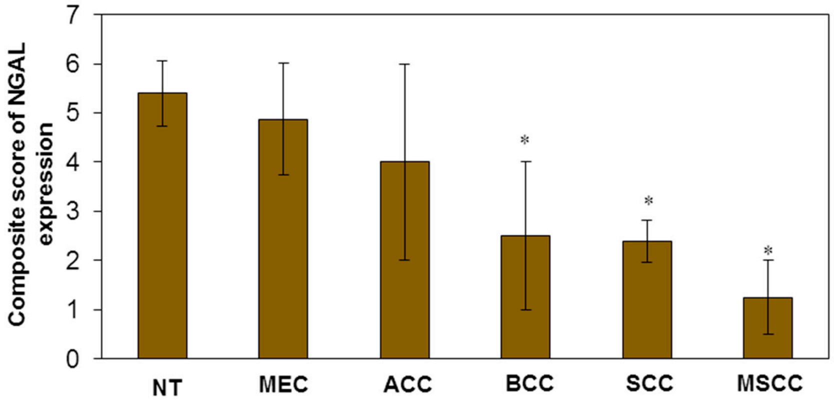

Figure S2. Expression of NGAL in different types of oral cancer. Mean composite score of NGAL levels in NT-Normal tissue ($n = 10$), MEC-Mucoepidermoid carcinoma ($n = 8$), ACC-Adenoid cystic carcinoma ($n = 3$), BCC-Basal cell carcinoma ($n = 2$), SCC-Squamous cell carcinoma ($n = 28$), and MSCC-Metastatic squamous cell carcinoma ($n = 4$) of oral cancer. Data are mean ± SE. * = $p < 0.05$.

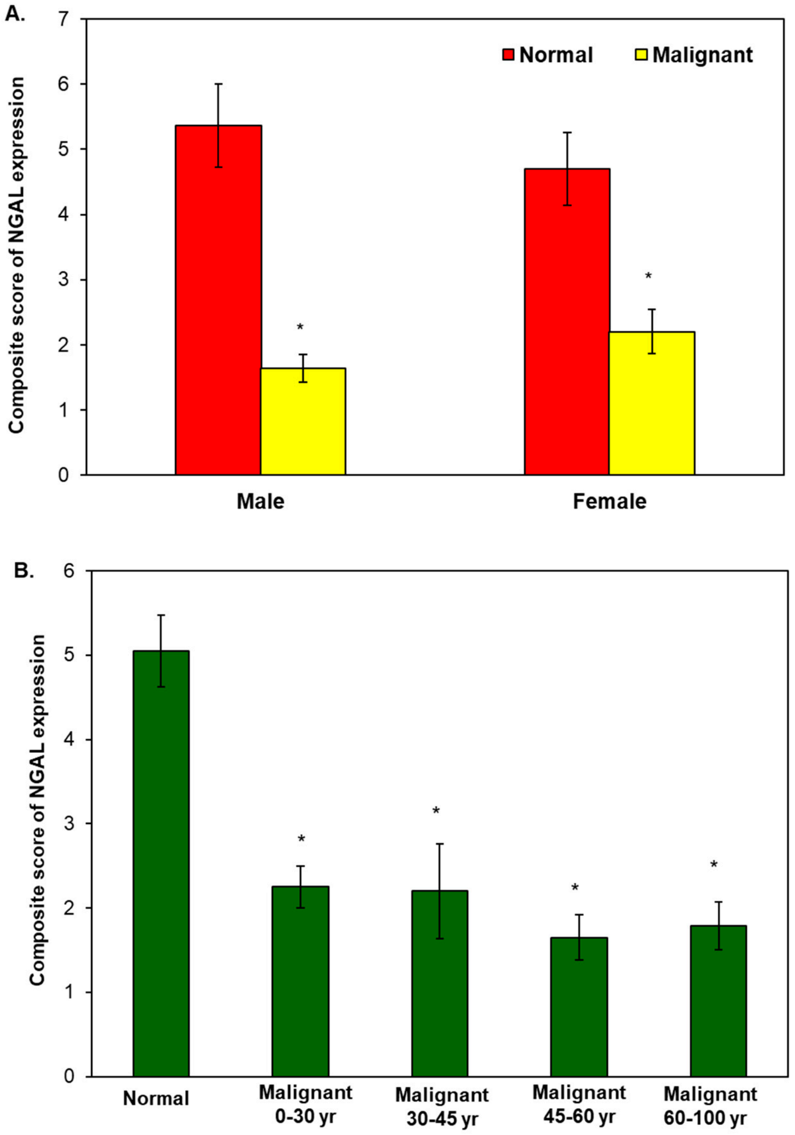

Figure S3. Expression of NGAL with age and gender in oral cancer tissues. (**A**) Mean composite score of NGAL levels in tissues from normal male (n = 11), normal female (n = 10), malignant male (n = 100), and malignant female (n = 39) patients with oral cancer. (**B**) Mean composite score of NGAL levels in normal (n = 17) and malignant oral cancer patient tissues of age groups 0–30 yr (n = 4), 30–45 yr (n = 18), 45–60 yr (n = 63), and 60–100 yr (n = 52). Data are mean ± SE. * = $p < 0.05$.

© 2018 by the authors. Licensee MDPI, Basel, Switzerland. This article is an open access article distributed under the terms and conditions of the Creative Commons Attribution (CC BY) license (http://creativecommons.org/licenses/by/4.0/).
